# Ethnic differences in the association between waist-to-height ratio and albumin-creatinine ratio: the observational SUNSET study

**DOI:** 10.1186/1471-2369-13-26

**Published:** 2012-05-07

**Authors:** Irene GM van Valkengoed, Charles Agyemang, Ray T Krediet, Karien Stronks

**Affiliations:** 1 Department of Public Health, Academic Medical Centre, University of Amsterdam, Amsterdam, the Netherlands; 2 Division of Nephrology, Department of Internal Medicine, Academic Medical Centre, University of Amsterdam, Amsterdam, the Netherlands

## Abstract

**Background:**

Ethnic differences in the association between central obesity and raised albumin-creatinine ratio (ACR) have not been investigated. Our aim was to determine whether the association between central obesity, defined by the waist-to-height ratio (WHtR), and ACR differed between subjects of Hindustani-Surinamese, African-Surinamese and Dutch origin.

**Methods:**

In total, 334 Hindustani-Surinamese (~South Asian), 589 African-Surinamese (~African), and 493 Dutch (~European) men and women, aged 35–60 years, randomly selected from the municipal register of Amsterdam, participated in an interview and physical examination.

We calculated the WHtR by dividing the waist circumference by height and the log ACR (logACR, log mg/mmol) by log-transforming the albumin concentration by the creatinine concentration in urine. The association between WHtR and logACR was studied in the total population and stratified by ethnicity. We also tested for interaction.

**Results:**

In the total population, a higher WHtR was associated with a higher logACR, after adjustment for sex, age, and smoking, body mass index and the presence of type 2 diabetes or hypertension. Among the Hindustani-Surinamese, the adjusted association between WHtR and logACR appeared somewhat stronger than among the other ethnic groups: for every 0.1 increase in the WHtR, the log-ACR increased by 0.522 (0.096-0.949) log mg/mmol among the Hindustani-Surinamese, by 0.334 (0.047-0.622) among the African-Surinamese and by 0.356 (−0.010-0.721) among the Dutch. However, the interaction was not statistically significant.

**Conclusions:**

WHtR was associated with a higher ACR among populations of Hindustani-Surinamese, African-Surinamese and Dutch origin. Our study seems to support global use of WHtR in relation to ACR across ethnic groups. However, although not significant, the association appeared slightly stronger among the Hindustani-Surinamese than among the other ethnic groups. If confirmed, this could have implications for use of the WHtR across ethnic groups.

## Background

An increased excretion of albumin in urine, often expressed as the albumin-creatinine ratio (ACR), is a risk factor for cardiovascular disease and an independent predictor of mortality in the general population [1,2]. Ethnic minorities have a greater risk of albuminuria and a higher ACR than subjects of European origin [3-6].

The higher prevalence of raised ACR and albuminuria among some ethnic groups may be related to a high prevalence of central obesity. Several studies have demonstrated that central obesity is associated with a higher ACR and albuminuria [7-12].

Besides a high prevalence of central obesity, the risk may also be related to differences in the association of central obesity with the ACR. This association could well differ across populations of various ethnicity, for instance due to differences in fat distribution [13]. However, although one study included subjects of diverse origin, no ethnicity-specific analyses were conducted [14].

Previous studies have mostly used the waist circumference or waist-to-hip ratio as a marker of central obesity. Recently, it has been suggested that the waist to height ratio (WHtR) should be used as global indicator for the health risks of obesity in (public health) practice [15,16]. The WHtR has been deemed a better, more sensitive measure for central obesity and is suggested to be superior for analysis of coronary risk and kidney disease [15-20]. One study has directly analyzed the association of WHtR with albuminuria in a European population with type 2 diabetes [19], but did not include ethnic minority populations.

Therefore, we aimed to investigate the association between waist-to-height ratio and the ACR in a population-based sample of 35–60 year old Hindustani-Surinamese (~South Asian), African-Surinamese (~African) and ethnic Dutch (~European) men and women, living in Amsterdam, the Netherlands. We specifically focused on potential differences in the association between ethnic groups.

## Methods

### Study population

The study population consisted of participants in the SUNSET study that is based on a random sample of 2975 Surinamese (i.e. with at least one parent born in Surinam) and Dutch individuals, aged 35 to 60 years of age, drawn from the Amsterdam population register [6,21]. In 1975, almost half of the population of the former Dutch colony Surinam migrated to the Netherlands. It is estimated that approximately 36% of these immigrants were Hindustani-Surinamese (originally from the Indian subcontinent) and 41% African-Surinamese (predominantly of African origin) [22].

Ethnicity was classified according to self-identification. If information on the self-identified ethnicity of the individual was lacking, information on the ethnic group of the mother, the father and the mother’s ancestors was used to classify participants.

### Data collection

Between 2001 and 2003, all subjects in the sample were approached for a face-to-face, structured interview by interviewers who had been matched by sex and origin. The interview included questions on self-identified ethnicity, migration history, demographic variables, lifestyle, and health status.

Immediately after the interview, participants were invited for a medical examination. Before the physical examination, trained physicians and research assistants verified that participants were fasting. During the examination, staff recorded: weight in light clothing on a SECA mechanical scale (SECA gmbh & co, Hamburg, Germany) to the nearest 200 grams; height to the nearest 0.01 meter by tape measure with participants standing against a wall, heels together, feet at a 45 degree angle and head positioned in the Frankfurt plane; waist circumference midway between the lower rib margin and the iliac crest and hip circumference at the maximum point over the greater trochanters to the nearest 0.01 meter by tape measure. After the subjects had emptied their bladder and had been seated for at least 5 minutes, blood pressure and resting heart rate measurements were obtained from each subject’s arm at heart level using an OMRON-M4 semi-automatic sphygmomanometer (Omron Healthcare Europe BV, Hoofddorp, the Netherlands) with an appropriate-sized cuff. All anthropometric measurements and blood pressure measurements were obtained twice and the means were used for analysis.

Fasting glucose concentrations (mmol/l; HK/Glucose-6-P dehydrogenase test; P800 analyser, Roche Diagnostics, Indianapolis, IN) were determined in samples obtained at the time of the physical examination. Finally, urinary albumin (mg/l; Jaffé method; P800 analyser, Roche Diagnostics) and creatinine concentration (mmol/l; Immune-turbidimetry; P800 analyser, Roche Diagnostics) were measured in an early morning urine sample, collected on the day of the physical examination. These measurements were carried out at the Laboratory of Clinical Chemistry of the Academic Medical Center of the University of Amsterdam.

### Response and participation

**Figure 1:**
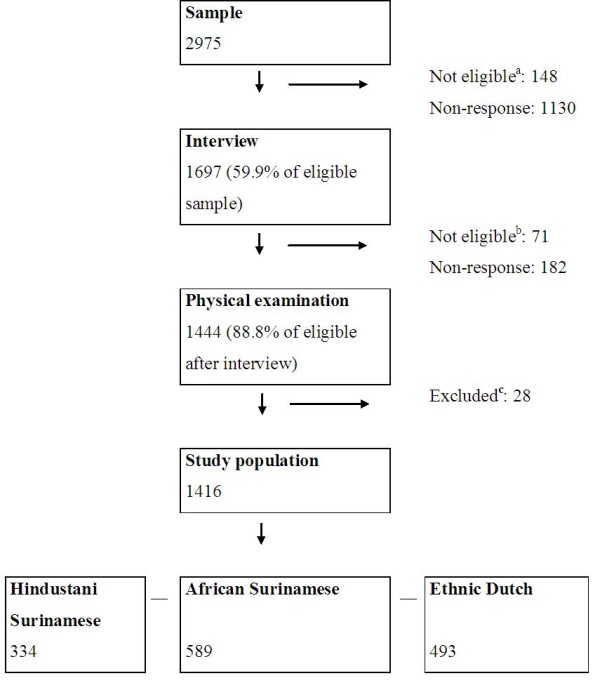
**Flow chart of the inclusion into the study. **^a^ Persons who had moved, were deceased or could not be reached at the registered address were not considered as potential participants; ^b^ Only persons of Hindustani Surinamese, African Surinamese and Ethnic Dutch origin were invited for the physical examination (n = 1626). Surinamese participants in the interview who originated from Java or China (i.e. ‘other’ ethnicity) and persons with missing ethnicity were excluded (n = 71); ^c^ Persons without a measured waist circumference, height, urine albumin and creatinine measurement were excluded (n = 28). The inclusion in SUNSET was reported previously [21].

The overall response to the interview was 60% (Figure 1). Participants in the interview were more likely to be female, married and living with a partner and/or children, and to live in an area with a density < 2500 addresses/km^2^. However, the differences between participants and non-participants were small and trends were similar across ethnic groups (data not shown). Of those who were eligible (n = 1626), 89% participated in the physical examination (Figure 1).

In the present analysis, we included subjects who had participated in the medical examination and had complete data on waist circumference, height, urine albumin and creatinine: 334 Hindustani-Surinamese, 589 African-Surinamese and 493 Dutch. As compared to those whose data were analyzed, those who did not undergo a physical examination or had incomplete data were similar with regard to sex, self-reported morbidity and self-rated health (data not shown).

### Definitions

First, we calculated the ACR by dividing the urine albumin concentration by the creatinine concentration (mg/mmol). Due to the skewed distribution, the resulting values were log-transformed before analysis (logACR). Because of the occurrence of albumin concentrations of 0 mg/l (n = 41), a constant of 0.005 was added prior to transformation and, therefore, no back-transformations could be performed. Microalbuminuria was defined as an ACR 2.5-30 mg/mmol for men and 3.5-30 mg/mmol for women and macroalbuminuria as an ACR ≥ 30 mg/mmol [23] Due to the relatively small numbers of persons with macroalbuminuria, these categories were combined for analysis.

Then, WHtR was determined by dividing the waist circumference (m) by the measured height (m). High WHtR was defined as a value greater than 0.50 [15].

Finally, we defined the body mass index by weight (kg) divided by height (m^2^); type 2 diabetes by fasting glucose ≥ 7.0 mmol/l and/or self-reported type 2 diabetes, excluding the self-reported diagnosis of gestational diabetes; hypertension by a SBP ≥ 140 mm Hg, or DBP ≥ 90 mm Hg, or being on anti-hypertensive therapy.

### Statistical analyses

Overall differences in characteristics between groups were assessed using Chi-square tests for categorical data and analysis of variance or Kruskal-Wallis tests for continuous measures. Then, post hoc tests were used to determine whether the characteristics of the Hindustani Surinamese and the African Surinamese differed from the characteristics of the Dutch.

Next, we used linear regression analysis to estimate the association between the WHtR and logACR in the total population. We adjusted for ethnicity and known determinants of ACR: sex, age, BMI, type 2 diabetes and hypertension. Moreover, we considered interaction by sex and age, as the association between body composition and chronic kidney disease has been reported to be age- and sex-dependent [24]. In addition, to verify the consistency of the associations found among subjects with and without pre-existent morbidity, we considered interaction due to the presence of hypertension or type 2 diabetes.

Subsequently, we examined the association of WHtR and logACR, stratified by ethnicity. We also tested formally for interaction between WHtR and ethnicity by adding a multiplicative interaction term to the fully adjusted model.

All analyses were performed using the SAS package, version 9.1 (SAS Institute Inc., Cary, NC). P-values ≤0.05 for the likelihood ratio test (≤0.10 for interaction), were considered statistically significant.

### Ethical approval

The SUNSET-study was approved by the Institutional Review Board of the Academic Medical Center, and carried out in accordance with the Helsinki Declaration. All participants provided a written informed consent.

## Results

### Characteristics of the study population

Dutch participants were older and had a higher level of education than Hindustani-Surinamese and African-Surinamese participants (Table 1). Among men, the African Surinamese smoked more frequently than the Dutch, while among women the Hindustani Surinamese and the African Surinamese smoked less frequently than the Dutch. Among women, but not men, the Dutch more frequently complied with the guideline for physical activity than the Hindustani Surinamese. Dutch women, but not Dutch men, had a lower mean BMI than the Hindustani-Surinamese and African-Surinamese. Type 2 diabetes (in men and women) and hypertension (women only) were more prevalent among Hindustani-Surinamese and African-Surinamese than Dutch participants.

**Table 1:** Characteristics of Hindustani-Surinamese, African-Surinamese and Dutch participants

**Men**			
	**Hindustani-Surinamese, N = 147**	**African-Surinamese, >N = 191**	**Dutch, N = 243**
**Mean age (years)**	44.5 (43.4-45.6)*	44.0 (43.2-44.9)*	**48.0 (47.1-48.8)**
**Highest level of education (%)**			
Primary or less	43 (29.7)*	15 (8.0)*	**20 (8.4)**
Secondary	**61 (42.1)**	**93 (49.5)**	**52 (21.9)**
Lower vocational	**27 (18.6)**	**49 (26.1)**	**69 (29.0)**
Higher vocational or more	**14 (9.7)**	**31 (16.5)**	**97 (40.8)**
**Smoking (current)**	**78 (53.1)**	111 (58.7)*	**109 (45.2)**
**Physical activity** (%)	89 (60.5)	107 (56.0)	160 (65.8)
**Body mass index (kg/m2)**	26.4 (25.5-27.2)	26.3 (25.7-26.9)	26.2 (25.7-26.8)
**Type 2 diabetes (%)**	37 (25.3)*	**23 (12.1)**	**19 (8.0)**
**Hypertension (%)**	52 (36.4)	81 (42.9)	86 (35.4)
**Mean waist-to-height ratio**	0.56 (0.55-0.57)*	0.52 (0.51-0.53)*	**0.53 (0.52-0.54)**
- Waist-to-height ratio >0.50 (%)	117 (79.6)*	98 (51.3)*	**155 (63.8)**
- Mean waist circumference (m)	**0.95 (0.93-0.97)**	0.91 (0.89-0.92)*	**0.96 (0.94-0.97)**
- Mean height (m)	1.70 (1.69-1.71)*	1.75 (1.74-1.76)*	**1.80 (1.79-1.81)**
**Median ACR (mg/mmol)**	0.36 (0.20-0.91)*	**0.26 (0.16-0.47)**	**0.24 (0.16-0.45)**
- Albuminuria (%)	15 (10.2)	11 (5.8)	12 (5.0)
**Mean logACR**	−0.70 (−0.89- -0.51)*	**−1.11 (−1.31- -0.92)**	**−1.34 (−1.50- -1.17)**
**Women**			
	**Hindustani-Surinamese, ** **N = 187**	**African-Surinamese , ** **N = 398**	**Dutch, ** **N = 250**
**Mean age (years)**	45.0 (44.1-46.0)*	43.5 (43.0-44.1)*	**48.0 (46.7-48.4)**
**Highest level of education (%)**			
Primary or less	44 (24.0)*	26 (6.6)*	**21 (8.4)**
Secondary	**82 (44.8)**	**148 (37.6)**	**74 (29.7)**
Lower vocational	**39 (21.3)**	**135 (34.3)**	**71 (28.5)**
Higher vocational or more	**18 (9.8)**	**85 (21.6)**	**83 (33.3)**
**Smoking (current)**	42 (22.8)*	124 (31.7)*	**1109 (44.0)**
**Physical activity**- ≥30 minutes, 5 times/week (%)	82 (44.1)*	**226 (56.8)**	**155 (62.0)**
**Body mass index (kg/m2)**	27.8 (27.0-28.5)*	29.0 (29.0-30.1)*	**26.1 (25.4-26.7)**
**Type 2 diabetes (%)**	49 (26.5)*	51 (12.9)*	**14 (5.9)**
**Hypertension (%)**	61 (33.0)*	137 (34.8)*	**42 (16.8)**
**Mean waist-to-height ratio**	0.60 (0.59-0.61)*	0.58 (0.57-0.59)*	**0.53 (0.52-0.53)**
- Waist-to-height ratio >0.50 (%)	163 (87.2)*	323 (81.2)*	**135 (54.0)**
- Mean waist circumference (m)	0.94 (0.92-0.96)*	0.95 (0.94-0.97)*	**0.88 (0.86-0.90)**
- Mean height (m)	1.58 (1.57-1.59)*	1.63 (1.62-1.63)*	**1.67 (1.67-1.68)**
**Median ACR (mg/mmol)**	0.48 (0.30-0.97)*	0.40 (0.24-0.76)*	**0.34 (0.20-0.59)**
- Albuminuria (%)	13 (6.9)*	27 (6.8)*	**4 (1.6)**
**Mean logACR**	−0.65 (−0.89- -0.42)*	−0.70 (−0.84- -0.56)*	**−1.12 (−1.27- -0.97)**

Data are median (IQR), mean (95%-confidence interval) or N (%). Bold = difference between ethnic groups p ≤ 0.05 (Chi-square tests for categorical data and analysis of variance or Kruskal-Wallis tests for continuous measures), * Significantly different from Dutch (post hoc test) ACR = albumin-creatinine ratio, logACR = log albumin-creatinine ratio, Albuminuria = ACR 2.5-30 mg/mmol for men and 3.5-30 mg/mmol for women (microalbuminuria), or ACR ≥ 30 mg/mmol (macroalbuminuria). Education: Primary or less = 6 years or less, Secondary = 10 years, Lower vocational = 11–12 years, Higher vocational or more = ≥15 years. Physical activity = at least ≥30 minutes, 5 times/week.

### Mean waist to height ratio and albumin-creatinine ratio

The mean WHtR among Hindustani Surinamese men and women and among African Surinamese women was higher than the mean WHtR among Dutch men and women (Table 1). Hindustani-Surinamese men had the highest median ACR of 0.36 (0.20-0.91), followed by the African-Surinamese 0.26 (0.16-0.47) and the Dutch 0.24 (0.16-0.45) mg/mmol (Table 1). The prevalence of albuminuria did not differ across the groups among men. Among women, the Hindustani-Surinamese and African-Surinamese women had a higher median ACR than the Dutch (median ACR of 0.48 (0.30-0.97), 0.40 (0.24-0.76) and 0.34 (0.20-0.59) mg/mmol, respectively). A similar pattern was observed for the prevalence of albuminuria.

### Association between waist-to-height ratio and logACR

In the total population, the logACR increased by 0.359 (0.278-0.440) for every 0.1 point increase in the WHtR (Table 2). The association remained similar after adjustment for sex, age, and smoking, BMI and the presence of type 2 diabetes or HT; the association was 0.368 (0.174-0.562). No significant interaction was observed for age and sex, and type 2 diabetes or hypertension (Table 3).

**Table 2:** The association between waist-to-height ratio and the log albumin-creatinine ratio

	**Model 1**		**Model 2**		**Model 3**		**Model 4**	
	**Dif**	**95%-CI**	**Dif**	**95%-CI**	**Dif**	**95%-CI**	**Dif**	**95%-CI**
**Waist-to-height ratio** (per 0.1)	**0.359**	**0.278-0.440**	**0.272**	**0.185-0.360**	**0.451**	**0.255-0.647**	**0.368**	**0.174-0.562**
**Ethnicity** Hindustani African Dutch			**0.47** **0.35** **Ref**	**0.28-0.67** **0.18-0.52**	**0.41** **0.36** **Ref**	**0.20-0.62** **0.19-0.53**	**0.23** **0.23** **Ref**	**0.02-0.43** **0.06-0.40**
**Sex** (male)			**−0.21**	**−0.36- -0.06**	**−0.20**	**−0.35- -0.06**	**−0.29**	**−0.44- -0.14**
**Age** (years)			**0.02**	**0.01-0.03**	**0.02**	**0.00-0.03**	0.00	−0.01-0.01
**Smoking** (current)			**0.21**	**0.06-0.35**	**0.20**	**0.05-0.35**	**0.21**	**0.07-0.36**
**Body mass index** (kg/m2)					**−0.03**	**−0.06-0.00**	**−0.04**	**−0.07- -0.01**
**Type 2 diabetes**							**0.72**	**0.50-0.93**
**Hypertension**							**0.35**	**0.32-0.67**

Dif = difference, Ref = reference, CI = confidence interval, Bold = p-value ≤0.05, Model 1 = Unadjusted, Model 2 = Model 1 plus ethnicity, age, sex, smoking, Model 3 = Model 2 plus body mass index, Model 4 = Model 3 plus type 2 diabetes, hypertension.

**Table 3:** Interaction between waist-to-height ratio and sex, age, type 2 diabetes, hypertension

			**Dif**	**95 %-CI**	**p-value interaction**
**Sex**	**WHtR**	(per 0.1)	**0.355**	**0.154-0.555**	
	**Sex**	(male)	−0.55	−1.52-0.43	
	WHtR *Sex		0.047	−0.131-0.225	0.60
**Age**	**WHtR**		**0.729**	**0.158-1.300**	
	**Age**	(years)	0.04	−0.02-0.11	
	WHtR *Age		−0.008	−0.020-0.004	0.19
**Type 2 diabetes**	**WHtR**	(per 0.1)	**0.383**	**0.186-0.579**	
	**Type 2 diabetes**		**1.45**	**0.00-2.89**	
	WHtR *Type 2 diabetes		−0.120	−0.354-0.114	0.32
**Hypertension**	**WHtR**	(per 0.1)	**0.334**	**0.133-0.535**	
	**Hypertension**		−0.15	−1.16-0.87	
	WHtR *Hypertension		0.115	−0.060-0.291	0.20

Dif = difference, CI = confidence interval, WHtR = waist-to-height ratio, bold = p-value ≤0.05 and p ≤ 0.10 for the interaction term, Model includes sex, age, ethnicity, smoking, body mass index, hypertension, type 2 diabetes and listed interaction term.

Among the Hindustani-Surinamese, the adjusted association between WHtR and logACR appeared stronger than among the other ethnic groups (Table 4). For every unit increase in the WHtR, the logACR increased by 0.522 (0.096-0.949) log mg/mmol among the Hindustani-Surinamese, by 0.334 (0.047-0.622) among the African-Surinamese and by 0.356 (−0.010-0.721) among the Dutch. However, the formal test for interaction provided no evidence for a differential association across ethnic groups (Table 5).

**Table 4:** The association between waist-to-height ratio (per 0.1) and log albumin-creatinine ratio, stratified by ethnicity

	**Model 1**		**Model 2**		**Model 3**		**Model 4**	
	**Dif**	**95%-CI**	**Dif**	**95%-CI**	**Dif**	**95%-CI**	**Dif**	**95%-CI**
**Hindustani-Surinamese**	**0.317**	**0.127-0.507**	**0.223**	**0.017-0.429**	**0.630**	**0.189-1.071**	**0.522**	**0.096-0.949**
**African-Surinamese**	**0.355**	**0.233-0.477**	**0.309**	**0.174-0.445**	**0.415**	**0.114-0.705**	**0.334**	**0.047-0.622**
**Dutch**	**0.258**	**0.120-0.396**	**0.252**	**0.107-0.397**	**0.417**	**0.062-0.772**	0.356	−0.010-0.721

Dif = difference, CI = confidence interval, Bold = p-value ≤ 0.05, Model 1 = Unadjusted, Model 2 = Model 1 plus age, sex, smoking, Model 3 = Model 2 plus body mass index, Model 4 = Model 3 plus type 2 diabetes, hypertension.

**Table 5:** Interaction between waist-to-height and ethnicity in the association with log albumin-creatinine ratio

		**Difference**	**95%-CI**	**p-value interaction**
**WHtR**	(per 0.1)	**0.340**	**0.107-0.573**	
**Ethnicity**	Hindustani	0.39	−0.92-1.71	
	African	−0.08	−1.14-0.97	
	Dutch	Ref		
WHtR*Ethnicity	Hindustani	−0.027	−0.258-0.205	0.70
	African	0.058	−0.134-0.250	
	Dutch	Ref		

Dif = difference, Ref = reference, CI = confidence interval, WHtR = waist-to-height ratio, bold = p-value ≤0.05 . Model adjusted for ethnicity, age, sex, smoking, body mass index, type 2 diabetes, hypertension.

## Discussion

### Main findings

Central obesity, defined by the WHtR, was associated with a higher ACR among a multiethnic population of men and women aged 35–60 years. The association was consistent across sex and age groups and was not accounted for by the potentially mediating effect of pre-existent morbidity.

The association seemed somewhat stronger among subjects of Hindustani-Surinamese origin than among subjects of African-Surinamese or Dutch origin, although formal testing of interaction provided no further evidence for a differential association across ethnic groups.

### Discussion of the findings

#### Association WHtR and logACR

Our main finding that an increase in WHtR was associated with a higher ACR, was consistent with previous work. For instance, the association between central obesity and ACR in the Dutch men and women was in line with previous studies among populations of European origin [9,10,25].

For Hindustani Surinamese, the findings were in line with a study that reported an association between waist-to hip ratio and albuminuria among a South Asian population without type 2 diabetes [11]. However, the results are in contrast another study that reported the lack of a univariate association between the waist circumference and microalbuminuria among South Asian origin subjects with type 2 diabetes in India [26]. The difference between this study and our study may be related to the fact that our study also included persons without type 2 diabetes. The difference might also be related to the WHtR possibly being a more sensitive measure than waist circumference [15,19]. WHtR also encompasses the adjustment to different statures and thus accounts for the negative correlation of height to certain metabolic risk factors [17,18,27,28].

The association between central obesity and ACR in the African Surinamese is in line with studies that showed an association between central obesity and microalbuminuria in populations of African origin, living in the US, Jamaica and South Africa [29-31].

#### Differences in the association between ethnic groups

No prior studies have directly compared the strength of the association between central obesity and ACR between ethnic groups. Although not statistically significant, we observed that the association between central obesity and albumin-creatinine ratio appeared slightly stronger among the Hindustani-Surinamese than among the other ethnic groups. The lack of significance may in part be related to the size of our study in combination with the fact that it was based on a relatively young and healthy general population sample. A possible difference in the association between ethnic groups is all the more striking, given that the we used the WHtR. The WHtR is recommended as a measure that can be used uniformly across different ethnic groups, including populations of Asian origin, as the division by height (in part) counteracts the higher risk associated with smaller waist circumferences in these populations [15].

Nevertheless, this measure of ‘central obesity’ may still reflect a very different underlying visceral versus peritoneal fat distribution. For instance, a higher amount of visceral adipose tissue, a difference in the accumulation of subcutaneous fat and a larger adipocyte size has been found among South Asians as compared to Europeans [32-34]. Additionally, the pathways through which central obesity is associated with kidney damage may also vary between the ethnic groups. For instance, a difference in association between adiponectin, a hormone secreted by adipose tissue, and insulin resistance, a known risk factor for albuminuria, has been observed between ethnic groups [35-38]. Moreover, among South Asians, it has been suggested that the association between central obesity and insulin resistance, is not mediated by intramyocelular lipids, while it may play a role among European populations [39].

### Limitations

Our study also has some limitations that should be discussed. Firstly, any associations found between WHtR and ACR have to be interpreted with caution. Due to the cross-sectional nature of the study, no causal inferences can be made.

Second, we used WHtR based on a double measurement of the waist circumference and height of participants as a marker of central obesity. This type of measure is often chosen in epidemiological studies, although several more complex methods are available to quantify central obesity, such as magnetic resonance images, computed tomography, and dual-energy X-ray absorptiometry [40-42]. However, these are relatively time-consuming and costly and thereby less suitable for epidemiological studies. Nevertheless, an imperfect marker may have led to underestimation of the associations. However, as the bias is expected to be similar across ethnic groups, the differences in the associations between groups are expected to remain.

Moreover, we used the ACR based on a single urine sample. It has been argued that the use of a single urine sample may be less accurate than a 24-hour urine collection. Nevertheless, the ACR from a single untimed specimen has been shown to correlate well with a 24-hour collection, and is more practical and less expensive than 24-hour collections for epidemiological studies [43-45].

## Conclusions

We found that an increased WHtR was associated with a higher ACR among 35–60 year old men and women of South Asian, African and European origin with and without pre-existent morbidity. The WHtR is said to be a measure that suitably captures the risk associated with central obesity across ethnic groups, and our study seems to support this. However, the association with ACR appeared slightly stronger among the Hindustani-Surinamese than among the other ethnic groups. This possibly differential association should be further evaluated, as it may possibly have implications for the use of the WHtR as a marker for central obesity in relation to renal outcomes in (public health) practice.

## Competing interests

The author(s) declare that they have no competing interests.

## Author’s contributions

IGMvV designed the study, carried out the analysis, interpreted the results and wrote the first draft, CA and RTK contributed to the interpretation of the data and the revisions of the article, KS contributed to the design of the study, the interpretation and writing of the manuscript. All authors approved the final version of the manuscript.

## Pre-publication history

The pre-publication history for this paper can be accessed here:

http://www.biomedcentral.com/1471-2369/13/26/prepub
